# Involvement of Metabolic Lipid Mediators in the Regulation of Apoptosis

**DOI:** 10.3390/biom10030402

**Published:** 2020-03-05

**Authors:** Piotr Wójcik, Neven Žarković, Agnieszka Gęgotek, Elżbieta Skrzydlewska

**Affiliations:** 1 Department of Analytical Chemistry, Medical University of Bialystok, 15-089 Białystok, Poland; piotr.wojcik@umb.edu.pl (P.W.); agnieszka.gegotek@umb.edu.pl (A.G.); 2 LabOS, Rudjer Boskovic Institute, Laboratory for Oxidative Stress, Bijenicka 54, HR-1000 Zagreb, Croatia; Neven.Zarkovic@irb.hr

**Keywords:** apoptosis, lipid mediators, phospholipids, ROS, oxidative stress, endocannabinoids

## Abstract

Apoptosis is the physiological mechanism of cell death and can be modulated by endogenous and exogenous factors, including stress and metabolic alterations. Reactive oxygen species (ROS), as well as ROS-dependent lipid peroxidation products (including isoprostanes and reactive aldehydes including 4-hydroxynonenal) are proapoptotic factors. These mediators can activate apoptosis via mitochondrial-, receptor-, or ER stress-dependent pathways. Phospholipid metabolism is also an essential regulator of apoptosis, producing the proapoptotic prostaglandins of the PGD and PGJ series, as well as the antiapoptotic prostaglandins of the PGE series, but also 12-HETE and 20-HETE. The effect of endocannabinoids and phytocannabinoids on apoptosis depends on cell type-specific differences. Cells where cannabinoid receptor type 1 (CB1) is the dominant cannabinoid receptor, as well as cells with high cyclooxygenase (COX) activity, undergo apoptosis after the administration of cannabinoids. In contrast, in cells where CB2 receptors dominate, and cells with low COX activity, cannabinoids act in a cytoprotective manner. Therefore, cell type-specific differences in the pro- and antiapoptotic effects of lipids and their (oxidative) products might reveal new options for differential bioanalysis between normal, functional, and degenerating or malignant cells, and better integrative biomedical treatments of major stress-associated diseases.

## 1. Introduction

Apoptosis, a mechanism of programmed cell death, is an essential physiological process that occurs from the beginning of the life of a multicellular organism. Apoptosis is crucial in growth and development, as well as the pathophysiology of aging and disease. Usually, cells that become unnecessary at a particular stage of development, possess an abnormal structure, or display metabolic disorders resulting from pathological processes, undergo apoptosis. However, the lack of apoptosis in cells with sublethal DNA damage may lead to neoplastic transformation, while the intensification of apoptosis is often observed in inflammatory or autoimmune diseases. Through the process of clonal deletion, apoptosis also plays a crucial role in the elimination of autoreactive leukocytes that would otherwise have an adverse effect on the other cells [1]. Due to the importance of apoptosis for proper functioning of the organism, there are many mechanisms involved in its regulation. Often, these mechanisms are based on the induction or inhibition of the activity of signaling proteins by exogenous factors, which act as membrane receptor agonists, antagonists, or intracellular modulators [2,3,4]. 

Reactive oxygen species (ROS) are now recognized to play an increasingly important role in regulating overall cellular metabolism, including apoptosis. The overproduction of ROS—often associated with exogenous factors—can lead to a shift in redox balance towards pro-oxidative reactions, which cause oxidative stress [5,6,7]. Consequently, ROS modify major bioactive macromolecules such as DNA, lipids, and proteins. If damaged by ROS, the structure and function of DNA changes, potentially stimulating the activation of the so-called “guardian of the genome”—p53 protein, which initiates the process of apoptosis [8]. In addition, lipid modifications lead to the generation of lipid mediators, which—independent of ROS—cause changes such as alterations to the structure of signaling and structural proteins. These alterations can lead to metabolic dysregulation, including modification of transcription factor activity and, consequently, can promote cell death [9,10,11]. In this way, ROS may be involved in the regulation of major apoptosis signaling pathways.

## 2. Signaling Pathways of Apoptosis

**Figure 1:**
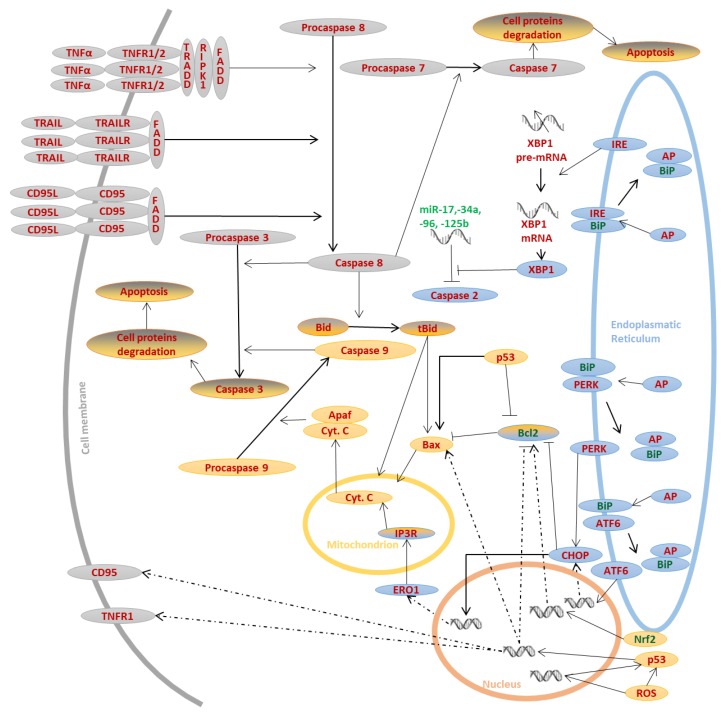
The main apoptotic pathways. Three main pathways are involved in apoptosis. Some molecules that regulate apoptosis may act as apoptosis activators (red) or repressors (green font). Abnormal proteins (AP) activate ER stress induced apoptotic pathway (blue) whereas death ligands attach to death receptors, which activates receptor pathway (grey). These two pathways may activate mitochondrial pathway (yellow) by the action of so-called molecular link-tBid protein. Nevertheless, mitochondrial pathway may be also activated independently.

Apoptosis is a precisely regulated process that can be initiated by both “death receptor” activation and metabolic changes in the cell [12,13]. In general, both proapoptotic signaling pathways coexist in any cell, and the activation of one pathway may result in the activation of the other because often, the same signaling factors are common elements of different metabolic pathways leading to apoptosis Figure 1.

### 2.1. Receptor Pathway

**Figure 2:**
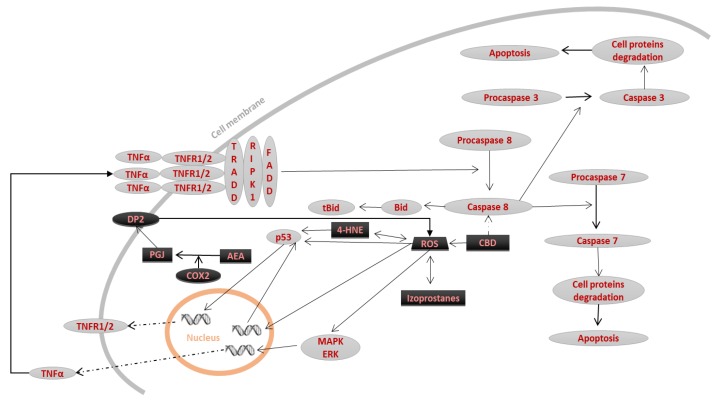
The influence of lipid mediators on receptor pathways. In receptor pathways, lipid mediators (black) act as activators of cell death. Their action leads to an increase in transcription of both death receptors, and death ligands which enhances apoptosis.

One of the primary mechanisms leading to cell apoptosis is the activation of death receptors through the attachment of an extracellular ligand Figure 2 [12]. Death receptors include receptors for tumor necrosis factor alpha (TNFα) (TNFR1 and TNFR2), receptors for TNF-related apoptosis-inducing ligand (TRAIL1R/DR4, TRAIL2R/DR5) and receptors for CD95L (Fas/CD95/APO-1). Expression of the receptors for TRAIL and CD95L is regulated by mitogen-activated protein kinase (MAPK), extracellular signal-regulated kinases (ERK1/2), and Fas-associated protein with death domain (FADD)—a protein necessary for the induction of apoptosis through the receptor pathway. Oxidative stress leads to the activation of these kinases, suggesting that it increases the sensitivity of the cell to apoptosis induced by death ligands. Therefore, exogenous antioxidants drive a reduction in apoptosis by reducing the levels of receptors and death ligands. Additionally, research has shown that ROS can activate the epidermal growth factor (EGF) receptor, which promotes the initiation of MAPK/ERK activity [14]. In contrast, the MAPKs p38 and c-Jun N-terminal kinase (JNK) are activated by various ligands, including apoptosis signal-regulating kinase 1 (ASK1). ASK1 is present in cells in a complex with thioredoxin (TRX), which is broken down by ROS, stimulating the activation of p38 and JNK [15]. JNK can also be activated by TNFR1 [16]. Activation of p38 and JNK increases TNFα transcription [2], and activation of p38 also leads to the increased expression of CD95L and p53, which induce apoptosis [17]. TNFα and ROS may also show specific positive feedback, because not only do ROS increase TNFα production, TNFα also increases ROS production. Additionally, TNFα is not only a ligand for TNFR, but also binds other receptors, such as Cdk5, which induces higher NADPH oxidase (NOX) expression, increasing ROS generation [18]. Moreover, complexes I and III of the mitochondrial respiratory chain are sensitive to TNFα, which enhances the increase in ROS production [19], which are capable of activating many apoptosis pathways directly related to the action of lipid mediators, so their excessive generation through activation of TNFR1 is an additional mechanism for the activation of apoptosis.

The activation of TNFR1 can lead to apoptosis or to the pro-inflammatory activation of inflammatory cells. After activation of TNFR1, the death domains are trimerized. This trimerization allows tumor necrosis factor receptor type 1-associated DEATH domain (TRADD) and receptor-interacting serine/threonine-protein kinase 1 (RIPK1) proteins attach to the death domains, and together form complex 1. The formation of complex 1 results in the activation of pro-inflammatory factors such as NFκB or JNK kinase [16]. If NFκB activation is impaired, FADD and procaspase 8 attach to TNFR1-associated complex 1. As a result, caspase-8 is activated through limited proteolysis, and the process of apoptosis begins [20,21]. 

The activation of TRAILR or CD95 receptors also results in the trimerization of the death domains, and the FADD adapter protein is attached directly to these trimers. This binding is necessary for the activation of procaspases 8 and 10, the initiator caspases [22]. Activated caspase-8—in addition to the activation of effector caspases (caspase 3 and 7)—can also, by limited proteolysis, convert the BID protein into tBID. This conversion acts as a signal to open channels in the mitochondrial membrane, enabling the release of cytochrome C from the mitochondria, which is the link between the receptor and mitochondrial pathways [10,22].

### 2.2. Mitochondrial Pathway

**Figure 3:**
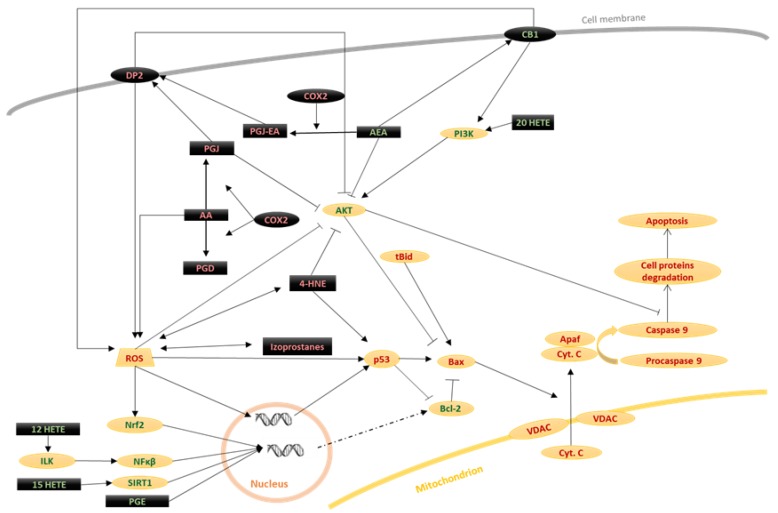
The influence of lipid mediators on the mitochondrial pathway. Large groups of lipid mediators (black) as well as other molecules associated with lipid metabolism and signaling (black) are involved in modulation of apoptosis. They can act as apoptosis activators (red font) or inhibitors (green font). Among activators, most important are products of COX-dependent phospholipid metabolism (mainly prostaglandins derivatives) and products of the non-enzymatic metabolism of phospholipids like 4-HNE or isoprostanes. On the other side, some lipids have a cytoprotective function, like anandamide or HETEs.

The second mechanism of activation of apoptosis involves the mitochondria. The mitochondrial pathway is primarily controlled by Bcl-2 family proteins, which include both antiapoptotic and proapoptotic factors. The antiapoptotic factors are proteins containing four BH domains (Bcl-2, Bcl-xL, Bcl-w, and Mcl-1). The proapoptotic proteins can be divided into two groups; those containing BH1, BH2, and BH3 domains (Bax and Bak), and those containing only the BH3 domain (tBid, Bim, Bad, Noxa, Puma, and Bmf) Figure 3 [23,24]. The activity and expression of proteins from the Bcl-2 family are regulated, among others, by p53 protein and the transcription factor Nrf2. Through its DNA binding domain, p53 promotes the transcription of proapoptotic Bcl family proteins (Bax) and inhibits transcription of antiapoptotic Bcl-2 [8,25]. In contrast, Nrf2 enhances the transcription of the anti-apoptotic Bcl-2 proteins [26,27]. 

The first critical feature in the activation of the mitochondrial pathway of apoptosis is the action of Bax and Bak proteins directly on the mitochondrial membrane. Antiapoptotic proteins are thought to be in a complex with the proapoptotic Bax and Bak proteins to block their action. Proapoptotic proteins containing only the BH3 domain promote apoptosis by combining with the antiapoptotic proteins, permitting the release of Bax and Bak (in the case of Bad, Noxa, Bik, and Bmf) or by downstream activation of free Bax and Bak proteins (tBid, Bim and Puma) [24,28]. p53 plays a crucial role in the induction of apoptosis, owing to its ability to participate in protein–protein interactions. p53 binds and deactivates antiapoptotic Bcl-2 family proteins, especially Bcl-xL, and promotes oligomerization and activation of Bax [8,25].

Active Bax and Bak proteins release proapoptotic factors from mitochondria through voltage-dependent anion channel (VDAC)-dependent or independent mechanisms. In the VDAC-independent mechanism, Bax and Bak proteins penetrate and generate pores in the mitochondrial membrane. These pores permit the release of proapoptotic factors, especially cytochrome C, as well as endonuclease G (endo G), apoptosis-inducing factor (AIF), SMAC/Diablo, and Omi serine proteases/HtrA2, from the mitochondria [23]. In the VDAC-dependent mechanism, activated Bax or Bak attaches to VDACs, which leads to the opening of VDAC channels, which also causes the release of the proapoptotic compounds, as in the VDAC-independent mechanism [29]. Studies targeting VDAC using siRNA knock-down demonstrated significant inhibition of apoptosis, suggesting that apoptosis occurs primarily due to the VDAC-dependent mechanism [30].

The most critical factor released from the mitochondria is cytochrome C, which, together with the cytosolic proteins Apaf and procaspase-9, forms the apoptosome. To prevent cell death, a complex consisting of the inhibitors of apoptosis Bcl-2/Bcl-XL joins the apoptosome and blocks its functions. This complex is inactivated by tBid, Bad, and Bik proteins, causing the activation of procaspase-9 [31]. Caspase 9 is an initiator kinase that activates the effector caspase 3. Caspase-9 is also able to convert Bid to its active form (tBid) by cleaving the peptide bond at the Asp59 position [31]. 

Mitochondria play an important role in cell apoptosis, not only because of their release of cytochrome C, but also because they are the primary source of ROS that arise as a result of the leakage of anion radicals from the respiratory chain. This ROS generation promotes the actions of oxidative processes, including oxidative modifications of cell components, leading to apoptosis. Comparison of wild-type and respiratory-deficient HeLa cells illustrates that wild-type cells produce far more reactive oxygen species than respiratory-deficient cells, promoting better survival, especially when oxygen levels are high [32]. Mitochondria not only generate ROS but are also particularly sensitive to their effects. Mitochondrial DNA is particularly susceptible to ROS modifications because they lack the repairing enzymes that protect the DNA from oxidation in the nucleus [32]. 

### 2.3. Endoplasmic Reticulum (ER) Stress-Induced Pathway

**Figure 4:**
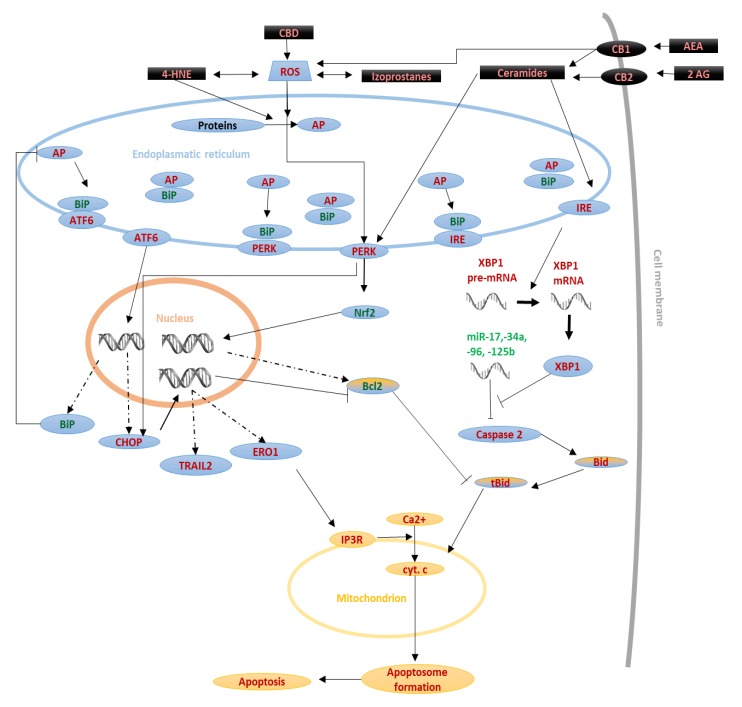
Influence of lipid mediators on ER stress-dependent pathway.Disorders in synthesis or action of exogenous factors, like lipid mediators (black) may lead to the generation and accumulation of abnormal proteins (AP). In consequence these proteins induce endoplasmic reticulum stress and activation of pro-apoptotic factors (red font). Moreover, ER stress leads to activation of mitochondrial pathway (yellow). On the other hand, during endoplasmic reticulum stress also some cytoprotective mechanisms are activated (green font).

Overproduction of ROS leads to the accumulation of lipid peroxidation products and oxidized proteins, which, like the accumulation of Ca^2+^ ions, promotes the induction of stress in the endoplasmic reticulum (ER) and activates the ER stress-induced apoptotic pathway. Consequently, a cascade of metabolic reactions leads to increased transport of calcium ions into the mitochondria, which can activate the mitochondrial pathway of apoptosis and thus promote cell death Figure 4 [33]. However, ER stress does not always cause apoptosis because many cytoprotective mechanisms are also activated to alleviate stress. For example, AKT kinase activation, and increased transcription of chaperones and Nrf2, which stimulate the transcription of antioxidant response element (ARE)-dependent cytoprotective genes such as MDM2 or Bcl-2 [26,34]. 

The endoplasmic reticulum is the site in the cell where protein synthesis and post-translational modifications occur. As a result, unfolded or damaged proteins can accumulate in this organelle. Such proteins have a high affinity for binding immunoglobulin protein (BiP), leading to the breakdown of the complexes binding BiP (i.e., BiP-PERK, BiP-ATF6, and BiP-IRE1α) [35]. This results in the release of IRE1α, PERK, and ATF6 from the BiP complexes, and their subsequent activation. Because IRE1α, PERK, and ATF6 are involved in the regulation of protein synthesis, their activation also leads to an increase in the expression of chaperones BiP and PDI, and the inhibition of mRNA translation, reducing the severity of endoplasmic stress [13,36]. Conversely, these proteins are also able to promote apoptosis, especially when ER stress is severe.

The action of IRE1α is characterized by both kinase and endo-RNAse activity [13]. The presence of unfolded proteins liberates IRE1α from the BiP complex. The release of IRE1α into an unbound state allows it to form oligomers, and its cytosolic domains are autophosphorylated, causing its activation. Activated IRE1α excises the regulatory intron from the X-box binding protein-1 (XBP-1) mRNA, which enables its translation [37]. XBP-1 degrades miRNAs-17, -34a, -96, -125b, which are repressors of caspase-2 translation. Therefore, XBP-1 translation leads to an increase in caspase-2, which, like caspase-8, converts Bid into tBid, eventually activating the mitochondrial pathway of apoptosis [38]. 

Activating Transcription Factor 6 (ATF6) also undergoes limited proteolysis under ER stress. Its cleaved N-terminal domain can then attach to ATF/cAMP response element (CRE) and ER stress-response elements (ERSE-1), stimulating expression of genes encoding for proteins such as BiP chaperone, proapoptotic C/EBP homologous protein (CHOP), and nuclear protein 1 (NUPR1) [39,40]. The protein kinase R (PKR)-like endoplasmic reticulum kinase (PERK) inhibits the phosphorylation of eukaryotic translational initiation factor 2 (eIF2α), inhibiting its activity. As a consequence, PERK impairs total protein synthesis in the cell, and there is no increased protein deposition in the ER. However, eIF2α phosphorylation also leads to increased CHOP and ATF4 transcription [41]. ATF4 increases GADD34 transcription, which dephosphorylates ATF6. Thus, its action leads to a negative loop [42]. ATF4 also promotes the transcription of other proteins from the ATF family, such as ATF3, and ATF5, but also CHOP, which is highly proapoptotic. Additionally, ATF4 increases the production of ROS, which can also be proapoptotic. The importance of ATF4 was demonstrated by blocking its transcription, which promoted apoptosis [43,44]. In contrast, translation of CHOP stimulates the transcription of protein phosphatase 1 regulatory subunit 15 (PPP1R15A/GADD34), TNF-related apoptosis-inducing ligand (TRAIL2), tribbles homolog 3 (TRB3), and, as a consequence, endoplasmic reticulum disulphide oxidase 1α (Ero1α). Finally, Ero1α stimulates the transport of Ca^2+^ to mitochondria via the IP3R receptor, causing cell death [45,46]. Notably, CHOP increases apoptosis not only by activating transcription of proapoptotic factors but also by inhibiting Bcl-2 transcription [36]. 

## 3. Participation of Phospholipid Metabolism Products in Apoptosis

**Figure 5:**
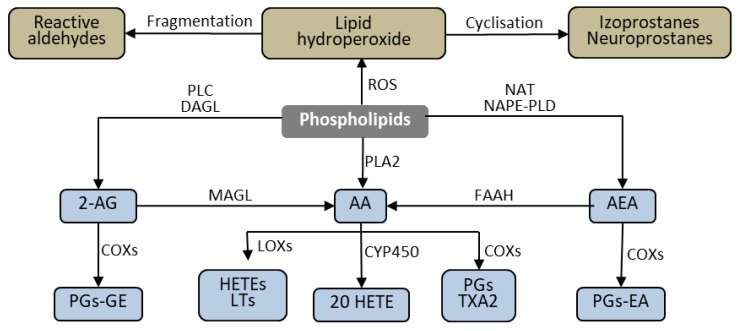
Phospholipid metabolism. 2-AG—2-arachidynoglycerol; 20 HETE—20 hydroxyeicosatetraenoic acid; AEA—anandamide; AA—arachidonic acid; COXs—cyclooxygenases; CYP450—cytochrome P 450; DAGL—diacylglycerol lipase; FAAH—fatty acid amide hydrolase; HETE—hydroxyeicosatetraenoic acid; LOX—lipooxygenasse; LTs-leukotrienes MAGL—Monoacylglycerol lipase; NAT—N-acyltransferase; NAPE-PLD—N-arachidonoyl phosphatidylethanolamine-preferring phospholipase D; PGs—prostaglandins; PGs—prostaglandin ethanolomides; PGs-GE—prostaglandin glycerol esters; PLC—Phospholipase C; TXA2- thromboxane A2.

Lipid mediators, produced from phospholipids, are critical influencers of apoptosis. Their generation is enhanced by the action of ROS [47]. Phospholipids are structural elements of biological membranes, and the lipid bilayer represents an important platform for proteins involved in cell signaling that affect intercellular communication, gene expression, and immune response [48]. However, under the influence of biological, chemical, and physical pathological factors, membrane phospholipids are metabolized to lipid mediators through ROS- and enzyme-dependent mechanisms Figure 5. These mediators—through various metabolic pathways—can modulate the process of apoptosis [47].

### 3.1. ROS-Dependent Lipid Peroxidation Products

Phospholipids containing polyunsaturated fatty acids (PUFAs), including arachidonic, linolenic, eicosapentaenoic, and docosahexaenoic acids, are particularly susceptible to ROS-dependent modifications [48]. These modifications are initiated mainly by hydroxyl or hydroperoxide radicals generated from superoxide, which is an essential product of cellular metabolism, including the respiratory chain reactions and the activities of NADPH and xanthine oxidases. Superoxide is metabolized by the antioxidant enzymes and transition metal ions to hydroperoxide and hydroxyl radicals, influenced by the intensity of cellular metabolism and the action of exogenous factors [49]. Pathological metabolism or increased activity of exogenous factors can result in increased ROS and the subsequent production of lipid peroxide radicals and lipid hydroperoxides.

Oxidative fragmentation of the alkyl chain of lipid hydroperoxides causes the formation of α,β-unsaturated reactive aldehydes, including 4-hydroxynenenal (4-HNE), 4-hydroxyhexenal (4-HHE), and malonic dialdehyde (MDA) [50,51]. Due to their electrophilic nature, these aldehydes have the ability to form complexes with the nucleophilic centers of proteins, phospholipids, and DNA, allowing them to they can modify their structure and functions. In this way, the formed aldehydes can participate in diverse cellular activities, such as transmitting signals in a variety of pathways [52,53,54]. 

One of the best-known products of ROS-dependent phospholipid metabolism is 4-HNE. 4-HNE contributes to the modeling of cellular signals, including those associated with the process of apoptosis. As evidence of this, research has demonstrated that 4-HNE can modify the structure of the MDM2 protein, breaking down the MDM2-p53 complex. Consequently, p53 is activated and translocates to the nucleus, where it stimulates the transcription of proapoptotic proteins, including Bax (responsible for the release of proapoptotic factors from the mitochondrion) and effector caspase-3 [55]. 

4-HNE may also interact with other proteins; for example, it forms adducts with His196, His267, Cys311, and Ser473 residues of AKT kinase, which results in a reduction in AKT sensitivity to phosphorylation. Additionally, modification of Ser473, considered to be the primary AKT regulatory site, leads to a decrease in the activity of the protein [56,57]. Because AKT has antiapoptotic effects through inhibiting several proapoptotic factors (including Acinus, AKS1, Bad, Bax, caspase-9) and activating antiapoptotic proteins (CREB and IKKα), suppression of AKT activity leads to a significant increase in apoptosis. Moreover, 4-HNE reduces the antioxidant capacity of cells by modifying glutathione (GSH) and GSH-Px structures, increasing oxidative stress [58]. These conditions favor the phosphorylation of MAPK ERK1/2, resulting in the activation of this pathway and increased apoptosis. Although the mechanism of this phenomenon is not fully understood, the contribution of oxidative stress is demonstrated by the finding that antioxidants partially abolish 4-HNE-induced apoptosis [59]. Similar to the proapoptotic activity of 4-HNE are the actions of another product of oxidative lipid fragmentation, 4-HHE [60]. However 4-HNE is also known for its dual functionality, as in low concentrations (below 10 μM), it can stimulate cell growth without significantly affecting apoptosis. To achieve these effects, 4-HNE must interact with EGF, as cells grown in conditions without EGF do not show a similar effect [61,62].

4-HNE and 4-HHE also strongly activate caspase-2 and caspase-3 and, to a certain degree, caspase-8. Inhibition of any of these caspases results in a significant reduction in the level of apoptosis, suggesting that each of them plays a significant role in apoptosis induced by 4-HNE and 4-HHE [60]. These findings indicate the involvement of 4-HNE and 4-HHE in the receptor pathway of apoptosis. Of particular relevance are the recent findings of novel anti-cancer, selective, concentration-dependent proapoptotic mechanisms of 4-HNE, through its inactivation of cancer-specific membrane-associated catalase [63]. In vitro, 4-HNE causes apoptosis selectively in NOX1-expressing tumor cells through the inactivation of membrane-associated catalase. Thus, 4-HNE reactivates subsequent intercellular signaling through the NO/peroxynitrite and HOCl pathways, followed by the mitochondrial pathway of apoptosis. Moreover, high concentrations of 4-HNE can induce both necrosis and apoptosis of tumor cells, while at lower concentrations, 4-HNE can activate amplificatory pathways based on singlet oxygen formation through hydrogen peroxide and peroxynitrite interaction, with activation of the FAS receptor and caspase-8. 

The anti-cancer pathways of 4-HNE may be useful for understanding its potential roles in the control of malignant cells and for the optimization of therapeutic approaches. In support of this is the recent finding that 4-HNE production is increased in non-malignant cells in the vicinity of human hepatocellular carcinoma or lung metastases of remote cancer. This highlights the possible role of 4-HNE as a natural anti-cancer substance as well as a detrimental factor in (neuro) degenerative and inflammatory processes [64,65,66,67,68]. Finally, the quantities of 4-HNE protein adducts are of high importance for the cytotoxic/apoptotic effects of 4-HNE since they represent a reservoir for the persistent presence of 4-HNE, even in the absence of oxidative stress [65,69]. The amount of 4-HNE protein adducts is age-dependent and negatively correlates with the amount of GSH [70,71]. Therefore, differences in GSH metabolism and overall antioxidant capacities between cancer cells and their non-malignant counterpart cells are crucial for the selective anti-cancer cytotoxic/apoptotic effects of 4-HNE, and for the oxidative stress-related anti-cancer effects of cytostatic drugs and other biomedical remedies [64,68,72,73,74,75].

Apoptosis induced by factors causing oxidative stress is often accompanied by an increase in the level of another lipid peroxidation product—MDA [76,77,78]. However, there is currently no evidence that MDA is directly involved in the process of apoptosis, although it might be involved in the stimulation of lipid metabolic pathways that affect apoptosis.

An alternative to the formation of reactive aldehydes is the intramolecular cyclization of lipid hydroperoxides. This causes the formation of prostaglandin derivatives, including isoprostanes (mainly from arachidonic acid) and neuroprostanes (mainly from docosahexaenoic acid), which are characterized by the presence of a cyclopentane ring [79,80]. Cyclic derivatives increase the production of ROS in mitochondria and further disturb the redox balance by oxidizing endogenous antioxidants, in particular, GSH [80]. The generated ROS can modify DNA, causing the oxidation of nitrogenous bases—especially guanine, which causes GA or GT conversion—and double-strand breaks, leading to the activation of the p53 protein, which initiates the mitochondrial pathway of apoptosis [81,82]. MAPK ERK1/2 also participates in isoprostane-induced apoptosis. Therefore, inhibition of MAPK ERK1/2 results in a significant reduction, but not complete inhibition, of isoprostane-induced apoptosis [80]. Isoprostanes interact with cells through several mechanisms, including by reacting with residues Phe196/184 and Asp193 on the thromboxane-like prostanoid (TP) receptors of which they are partial agonists [83]. The activation of TP receptors by their full agonists causes the inhibition of apoptosis [84]. However, it is not clear yet whether isoprostanes, as partial agonists, stimulate or inhibit the process of apoptosis through reactions with TP. Thus, the mechanisms of interaction of lipid peroxidation products in the process of apoptosis remain an open question.

### 3.2. Enzymes-Dependent Lipid Metabolism Products

#### 3.2.1. Eicosanoids

Regardless of lipid peroxidation, the enzymatic oxidation of fatty acids is a constantly ongoing cellular process that serves various functions. The products of arachidonic acid metabolism play a particularly important biological role. Arachidonic acid metabolism is catalyzed by cyclooxygenase (COX) and lipoxygenase (LOX). As a result of oxidation of arachidonic acid by COX, a group of prostanoids is formed, while in LOX-catalyzed reactions, leukotrienes, as well as hydroxyeicosatetraenoic acid (HETE), are generated Figure 5 [85]. The resulting compounds, especially those belonging to the eicosanoids: prostaglandins, leukotrienes, thromboxanes, and HETE derivatives show multidirectional effects, mainly associated with the regulation of immune cell function and the modulation of inflammation. Prostaglandins and leukotrienes primarily cause vasodilation and are also activators and chemoattractants for mast cells and eosinophils (prostaglandins) and lymphocytes (leukotrienes) [86,87,88]. HETE derivatives are neutrophil chemoattractants [89], and thromboxanes cause platelet aggregation and affect interactions of dendritic cells with lymphocytes, involving E- and D-series resolvins, and protectins arising from eicosapentaenoic acid (EPA) and docosahexaenoic acid (DHA) that play vital roles in suppressing inflammation [90].

In apoptosis, the role of eicosanoids is not entirely understood, although their ability to modulate the three primary pathways of apoptosis has been demonstrated. However, activation of the mitochondrial pathway may be secondary to both the receptor pathway and the pathway associated with ER stress, and these pathways can be activated both simultaneously and independently. This significantly hinders the unambiguous determination of the molecular mechanisms involved, especially for molecules involved in many different metabolic processes, such as eicosanoids. For some eicosanoids, it is even unclear whether their role is unambiguously pro- or antiapoptotic. It seems that this is influenced by cell type; the diverse distribution of both kinases and receptors in different cells results in differential involvement of eicosanoids.

Prostaglandins can increase apoptosis by enhancing the production of ligands for death receptors, which stimulates the apoptosis receptor pathway. In the case of pro-inflammatory prostaglandins from the J-series, it has been demonstrated that in keratinocytes, the responsible ligand is TNFα. This was illustrated using TNFα siRNA knock-down and inhibition of TNFR1 and TNFR2 receptors by their antagonists. Both of these approaches caused the inhibition of Prostaglandin J (PGJ)-induced apoptosis. The authors suggest that this effect occurs as a result of the activation of the D2 prostanoid receptor (DP2) by agonists such as 15d-PGJ2, which causes an increase in ROS production and the activation of MAPK p38, p42, p44, and MAPK ERK1 and MAPK ERK2, increasing TNFα transcription [2]. D-series prostaglandins (PGDs) have also been shown to activate the receptor pathway in chondrocytes, which could also depend on the stimulation of TNFα production [91]. This suggests that PGDs are also agonists of the DP2 receptor and that stimulation of the receptor pathway by PGD2 does not occur in osteoclasts, cells resistant to TNFα proapoptotic activity [92,93].

The J-series prostaglandins are also able to induce stress in the endoplasmic reticulum and thus stimulate apoptosis. PGJ can increase CHOP protein expression in cancer cells, and consequently, initiate a decrease in transcription of the antiapoptotic Bcl-2 protein [94]. This likely impacts the other consequences of ER stress. PGD2 has a similar effect, as ER stress induction has been demonstrated as a consequence of DP2 receptor activation [95]. The involvement of prostaglandins in the induction of ER stress also causes an increase in COX-2 expression, which is involved in the synthesis of prostaglandins [96]. This points to the existence of a positive feedback loop between prostaglandins and ER stress.

Furthermore, activation of the DP2 receptor also leads to the activation of apoptosis via the mitochondrial pathway. DP2 agonists such as PGJ2 and PGD2 cause a reduction in the antiapoptotic activity of AKT (measured as the level of its phosphorylation) and, consequently, an increase in the level of apoptosis [97,98]. This increase is mediated by—among other factors—an increase in Bax protein expression [98,99] which, by acting on mitochondria, releases proapoptotic factors, especially cytochrome C. However, in Th2 lymphocytes with IL-2 deficiency, DP2 agonists such as PGD2 and 13,14-Dihydro-15-keto-PGD2 (DK-PGD2) have been shown to act as antiapoptotic factors by activating phosphoinositide 3-kinase (PI3K), an antiapoptotic AKT activator [2,84]. However, it appears that the antiapoptotic effect of PGD2 is limited to IL-2-deficient Th lymphocytes. Eicosanoids such as 20-HETE are also able to activate PI3K kinase, and consequently increase AKT expression, which results in inhibition of the process of apoptosis [100]. Eicosanoids, including PGE [101,102], 15-HETE [103], 12-HETE [104], are capable of increasing the expression of Bcl-2, a protein that strongly inhibits the mitochondrial pathway. Therefore, these eicosanoids may also exert antiapoptotic effects on the mitochondrial pathway. In the case of PGE2, the stimulation of Bcl-2 expression occurs due to the activation of the E-type prostanoid receptor (EP2). The activation of EP2 reduces the expression of p53, one of the most important activators of apoptosis, which works through several mechanisms, including inhibiting Bcl-2 expression and activity [102]. 15-HETE and 12-HETE can also indirectly act on Bcl-2 by stimulating the expression of transcription factors that regulate Bcl-2, including sirtuin 1 (SIRT1) and integrin-linked kinase (ILK) [103,104].

In pulmonary epithelial cells, 20-HETE acts by activation of NADPH oxidase, slightly increasing the level of ROS that, surprisingly, exerts an antiapoptotic effect. Thus, it has been suggested that the use of anti-oxidants might abolish the protective effect of 20-HETE on cells. Indeed, a slight increase in the level of ROS causes activation of cytoprotective pathways (including those associated with Nrf2), without causing oxidative damage to the cells, which would lead to the activation of proapoptotic pathways [105]. Although the exact mechanisms of prostaglandin action in vivo have not been determined, PGDs are known to reduce tumor cell survival, while animal studies have shown that mice null for lipocalin-type prostaglandin D synthase (L-PGDS^-/-^) have significantly reduced levels of tumor cell apoptosis [106]. However, it has yet to be elucidated which apoptosis pathways are involved in this process.

Therefore, it seems that prostaglandins of the J- and D- series are proapoptotic in most cells, and this effect is strongly dependent on DP2 receptor activation, and at least partially on the reduction in AKT kinase activity. In contrast, prostaglandins of the E-series and HETE appear to act contrariwise to prostaglandins of the J- and D-series, reducing the level of apoptosis through the activation of AKT and increasing Bcl-2 expression.

#### 3.2.2. Endocannabinoids

As a result of the metabolism of phospholipids by enzymes from the phospholipase family, a group of lipid mediators—endocannabinoids are generated. These endocannabinoids are comprised of ester, and amide derivatives of long-chain PUFAs, especially arachidonic acid. The most numerous group of endocannabinoids consists of ethanolamides of fatty acids, including anandamide (AEA), which are synthesized from phospholipids located in the outer layer of the cell membrane (phosphatidylethanolamine and phosphatidylcholine). Derivatives of glycerol and fatty acids are another group of endocannabinoids, of which 2-arachidonoylglycerol (2AG) is the best known. The biological activity of endocannabinoids occurs mainly through the activation of G protein-related receptors, including CB1/2 cannabinoid receptors [107].

AEA is believed to stimulate apoptosis through the receptor pathway by activating CB1 or CB2 receptors, and activation of CB1 also stimulates the mitochondrial pathway. The ability of AEA to activate apoptosis through these receptors has also been observed in dendritic cells [108]. AEA enhances apoptosis by both activating initiator caspase-9 and executive caspases-3 and-7, a mechanism observed in chorionic cells (BeWo) [108]. Anandamide can also activate caspase-8, and the authors posit that both CB1 and CB2 receptors could be involved in this process [108]. However, it has also been suggested that MAPK p38 may be responsible for the activation of caspases by AEA, because its inhibitors abolish this effect in the endometrial cells of rats [109]. Importantly, the inhibition of the CB1 receptor, or the addition of an antioxidant such as N-acetylcysteine (NAC), causes only partial inhibition of apoptosis, as demonstrated in human coronary epithelial cells [110], while dual-treatment of rat endometrial cells with CB1 inhibitor and NAC almost completely blocks MAPK p38 activation and endocannabinoid-induced apoptosis [109]. This implies that the mechanisms associated with CB1 receptor activation, like ROS overproduction and ROS-dependent activation of MAPK p38, are responsible for the proapoptotic effect of endocannabinoids [109,110].

Endocannabinoids also modulate apoptosis by regulating antiapoptotic AKT [111]. However, the exact mode of such endocannabinoid action is not entirely clear. Endocannabinoids have been shown to reduce AKT expression, while activation of the CB1 receptor causes PI3K phosphorylation, which further phosphorylates AKT. Therefore, PI3K is thought to be involved in the activation of AKT by endocannabinoids because its inhibitors diminish this effect [112,113].

Endocannabinoids could also participate in apoptosis by inducing autophagy and ER stress, as the consequence of activation of cannabinoid receptors is an increase in ceramide production, which can lead to ER stress. This confirms the principle of ER stress markers, such as PERK phosphorylation, that increase in IRE1 activity and ATF6 translocation to the cell nucleus after the administration of endocannabinoids [47,114,115].

Endocannabinoids also interact with hypoxia-inducible factor 1 (HIF1) while inducing apoptosis. Under conditions of homeostasis, HIF1 is rapidly degraded, while overproduction of endocannabinoids promotes its stabilization, enhancing the attachment of HIF1 to the MDM2-p53 complex and its subsequent degradation. The degradation of this complex results in the activation of the proapoptotic factor p53, and the induction of mitochondrial apoptosis pathway [116]. Stabilization of HIF1 may also promote a feedback loop, resulting in reduced expression of the anandamide degrading enzyme fatty acid amide hydrolase (FAAH). FAAH may elevate the levels of endocannabinoids (especially anandamide) and, consequently, stabilize HIF1. In contrast, CB1 receptor antagonists block the proapoptotic effect of HIF1, which further suggests that under the influence of HIF1 stabilization, endocannabinoid levels increase [117].

There is also evidence that endocannabinoids can elicit cytoprotective effects. The cardioprotective drug propofol has been found to cause a significant increase in the release of endocannabinoids (AEA and 2-AG) by cardiomyocytes. This is accompanied by increased activation of CB1 and CB2 receptors at both mRNA and protein levels. In support of these findings, the cytoprotective effect of propofol is abolished by CB2 antagonists, although not by CB1 antagonists [118]. An effect similar to propofol is exhibited by an FAAH inhibitor (URB597) and an endocannabinoid reuptake inhibitor (VDM11) [118]. In addition, the exposure of neurons to another endocannabinoid—oleoylethanolamine (OEA)—also exerts a neuroprotective effect, in a manner dependent on the PPARα receptors of hypoxic neurons, which would otherwise cause their apoptosis, in part by increasing Bax expression and decreasing Bcl-2 levels [119,120].

In the context of these findings, the observation that endocannabinoids act differently on different cell populations seems to be essential in understanding the effects of endocannabinoids on apoptosis. These cell-type specific differences can be associated not only with different expression of receptors, but also with differences in the levels of endocannabinoid metabolic pathways in different cells. The main AEA and 2-AG metabolizing enzymes are FAAH and monoacylglycerol lipase (MAGL), respectively, which degrade endocannabinoids to arachidonic acid [121,122]. Other enzymes significantly involved in endocannabinoid metabolism are COX and LOX, which metabolize endocannabinoids to eicosanoids [123]. Differences in the expression of these enzymes cause different levels of endocannabinoid metabolites that mediate many cellular processes. COX-2 metabolizes 2-AG to prostaglandin glycerol esters (PG-GE), which are proapoptotic. Consequently, hematopoietic stem cells with high COX-2 expression undergo increased apoptosis when incubated with 2AG in vitro. Additionally, hepatocytes with low COX-2 expression do not undergo apoptosis after the administration of 2AG [124]. Similarly, in COX-2^-/-^ cells where PG-GE is not produced, there is no increase in apoptosis after 2-AG administration [124]. 

As in the case of AEA, when 2-AG is added to the culture of squamous cell carcinoma, the level of PGJs in the post-culture medium is increased [125]. The key role of COX-2 in the induction of apoptosis after the addition of endocannabinoids in vitro has also been demonstrated in HaCaT keratinocytes, in which the transfection of cells with a plasmid containing COX-2 cDNA caused the induction of ER stress and apoptosis following AEA treatment. The contribution of COX-2 in the metabolism of AEA to J series prostaglandin derivatives (15-deoxyΔ12.14PGJ2-EA, PGJ2-EA, and Δ12PGJ2-EA) has also been associated with the induction of apoptosis [47]. The presence of PGJ2 derivatives in post-culture media indicates the possibility of not only autocrine but also of paracrine action of the above-listed metabolites. This confirms the observation that ER stress appears not only in cells transfected with COX-2, but also in cells localized near them [47]. In the case of cancer cells with constitutively high COX-2 expression, inhibiting the activity of the AEA degrading enzyme FAAH enhances AEA metabolism involving COX, leading to an increase in the production of J-series prostaglandins and apoptosis [125]. In the apoptosis resistant HCT116 Bax -/- colon cell line, which is also extremely resistant to anti-cancer drugs, anandamide administration was able to induce apoptosis. In this case, COX-2 inhibition abolished this effect, which appears to be receptor independent [126].

#### 3.2.3. Exogenous Cannabinoids 

Not only endogenous cannabinoids can affect the process of apoptosis. Cannabinoids from different origins, including plants, can also influence apoptosis. Namely, the phytocannabinoid from *Cannabis sativa*, cannabidiol (CBD), which is not psychoactive, can modulate the process of apoptosis. The direction of action of CBD is dependent on the type of cells treated. In the case of fully differentiated cells such as neurons or cardiomyocytes, CBD protects cells against oxidative stress and apoptosis [126,127]. However, CBD intensifies oxidative stress and apoptosis in immune and cancer cells [128,129,130]. In the case of leukemic cells, CBD enhances oxidative stress by increasing the mRNA and protein levels of oxidative NOX4 and p22phox [128]. However, in the case of glioblastoma cells, CBD induces oxidative stress by reducing the activation of antiapoptotic AKT, which increases the level of apoptosis [131]. CBD also intensifies tBID translocation to mitochondria, which results in the release of cytochrome C, PARP fragmentation, and an increase in apoptosis [132]. Finally, the induction of apoptosis by CBD is associated with the activation of caspases 8 and 9 and an increase in the level of CHOP protein and activated PERK. This suggests that CBD works by modulating the three pathways leading to apoptosis: receptor, mitochondria and ER-associated [128,133,134], whereby activation of the mitochondrial pathway may be secondary to activation of the receptor or associated with ER stress. This is highly similar to the action of endocannabinoids, which also activate these pathways. However, in some cells, they act in a proapoptotic manner, and in others, act in an antiapoptotic manner [108,114,115,119,120].

The diverse effects of CBD on cells can be explained by differences in receptor expression in different cells. For example, in the case of neurons, the CB1 receptor dominates; whereas, in the case of tumors, the CB2 receptor is usually dominant [123]. This could indicate that the CB2 receptor is responsible for the proapoptotic activity of CBD, which is confirmed by evidence that inhibition of CB2 abolishes the proapoptotic activity, at least in the case of breast cancer cells [135]. In contrast, the protective effect of CBD appears to be independent of any receptors because CB1, CB2, transient receptor potential cation channel subfamily V member 1 (TRPV1), and peroxisome proliferator-activated receptor γ (PPARγ) antagonists do not affect the CBD pro-survival effect [136]. It cannot be excluded that the antiapoptotic function of CBD results from the action of other receptors, for example, from the PPAR family. The fact that the proapoptotic effect is induced by receptor activities, while the protective effect appears to be independent of receptors, suggests that increased expression of cannabinoid receptors, especially CB2, determines the cell’s sensitivity to CBD-mediated apoptosis. 

In lung cancer cells, CBD increases the levels of proapoptotic 15d-PGJ2 and PGD2 [137]. Since inhibition of COX-2 results in a reduction in 15d-PGJ2 and PGD2 levels, COX-2 could significantly reduce the proapoptotic activity of CBD in these cells through this mechanism [137]. This would suggest that CBD itself, like endocannabinoids, is in antiapoptotic, while the induction of apoptosis by CBD in some cells is coupled to lipid mediators produced during COX-2-dependent CBD metabolism. This would suggest that CBD promotes apoptosis of cells involved in inflammation and cancer because they have high COX-2 activity. The ability of CBD to induce apoptosis was also observed when treating refractory cancers, such as the gastric cancer line SGC-7901. In these cells, CBD induced growth cycle arrest and stimulated apoptosis by activating oxidative stress [138].

Apoptosis is modulated by other lipid mediators, such as ceramides, the level of which may be modified by another phytocannabinoid—tetrahydrocannabinol (THC). The addition of THC to cells, such as dendritic cells, leads to apoptosis, whereas the use of CB1 or CB2 receptor inhibitors prevents apoptosis. Both caspases 8 and 9 are overexpressed after the administration of THC, suggesting that THC-mediated apoptosis results from both mitochondrial and receptor pathways [139]. It has also been shown that THC increases the accumulation of ceramides in cells, while inhibition of their synthesis abolishes the proapoptotic effect of THC. This implies that the accumulation of ceramides under the influence of THC is the reason for the proapoptotic action of THC [140]. An increase in ceramide production also induces ER stress and apoptosis, resulting from the activation of CB1/CB2 receptors by THC [141]. Moreover, the increase in ceramide synthesis under the influence of THC is accompanied by an increase in eIF2α phosphorylation and, consequently, an increase in the expression of NUPR1 and TRB3. Importantly, inhibition of de novo ceramide synthesis significantly reduces the expression of ER stress markers, confirming the effect of ceramides on the process of ER stress and apoptosis. Finally, ceramide analogs also increase cell sensitivity to CD95L receptor-induced apoptosis [141]. 

In ceramide-induced apoptosis—the activator of the mitochondrial apoptotic pathway—is formed, enhancing the release of cytochrome C from mitochondria [22]. In vitro studies have shown that the activity of sphingomyelinases, enzymes involved in the formation of ceramides, is necessary for the release of cytochrome C from mitochondria. Furthermore, blocking the activity of other enzymes involved in ceramide production also results in inhibition of cytochrome C release from mitochondria [142]. Ceramides also enhance apoptosis through the MAPK ERK1/2 pathway, thus showing some similarity to aldehydes formed as a result of ROS on lipids like 4-HNE [109,143]. THC also works in this way in treatment-resistant glioblastoma cells, in which THC administration leads to apoptosis of these cells. In this case, inhibition of ceramide synthesis prevents apoptosis [144]. However, some glioblastoma cancer lines are resistant to THC-induced apoptosis. It has been suggested that the midkine/ALK axis plays an essential role in this. The midkine/ALK axis usually inhibits autophagy, but after THC induction, cells become sensitized to apoptosis [144].

It seems that ceramides also play an essential role in ionizing radiation-induced apoptosis. Acid sphingomyelinase is translocated into the cell membrane, increasing de novo ceramide synthesis (especially of C16 and C24 ceramides). Similarly, the addition of exogenous C16 ceramide or sphingomyelinase has been found to increase apoptosis, while inhibition of sphingomyelinase suppresses apoptosis [145]. 

### 3.3. Cross Talk between Lipids, Glucose, and Glutamine

Higher levels of both lipids and glucose are typical metabolic changes in diabetes and metabolic syndrome. However, increased levels of fatty acids, especially in the presence of glucose, increases ROS production, which ultimately leads to cell death [146]. However, inhibition of some enzymes involved in lipid metabolism, such as acyl-CoA synthase, protects cells from fatty acid-induced apoptosis under elevated glucose conditions, suggesting that it is not the lipids, but their metabolites, that are responsible for this reaction [147]. Despite the suggestion that elevated levels of fatty acids or glucose are not toxic, prolonged exposure to high levels of glucose and fatty acids would cause the accumulation of free fatty acid-derived long-chain acyl-CoA esters (FACoAs) and various lipid signaling molecules in cells, causing apoptosis [148]. Lipids are able not only to modify the function of local cells but also because lipid mediators activate JNKs, can inhibit transcription of insulin [149], which is a survival protein, and its absence causes increased apoptosis in the human organism [150,151]. On the other hand, insulin can be glycated when the glucose level is high, which causes the protein to become toxic to cells, which prevents β-Hydroxybutyric acid (BHB) production [151]. BHB also blocks apoptosis by reducing MAPK p38 and MAPK JNK activity, as well as generating reactive oxygen species [152]. 

Increased metabolism of glutamine to glutamate is observed in cancer cells. Glutamate is then converted into α-ketoglutarate and incorporated into the tricarboxylic acid (TCA) cycle, which leads to the production of the energy necessary to carry out cellular processes [153]. However, stopping this process is not sufficient to cause cancer death. This is probably due to this pathway being compensated by increased lipid metabolism [154]. 

## 4. Conclusions

During the metabolic reactions in the cell, including phospholipid metabolism, there is a continuous formation of molecules that mediate intracellular and systemic signals belonging to ROS/enzyme-dependent phospholipid metabolites. Most products of phospholipid metabolism enhance the process of apoptosis. However, their effects on apoptosis depend on the type of cells in which they are acting. Differences between cell types in the pro and antiapoptotic action of phospholipids and their derivatives may open up new possibilities for differential bioanalysis between healthy, functional, and degenerative or malignant cells. Therefore, science faces the need to determine the cases—depending on the type of cells involved—in which therapeutic strategy should be used. This could represent proapoptotic effects, in the case of cancer [155,156], or antiapoptotic effects, for example, in the protection against neurodegenerative disorders [157]. Given the possibility of the modification of cell phospholipid metabolisms by exogenous compounds, there are real possibilities of modulating the cell death process, depending on the needs of therapy.
